# A universal genomic coordinate translator for comparative genomics

**DOI:** 10.1186/1471-2105-15-227

**Published:** 2014-06-30

**Authors:** Neda Zamani, Görel Sundström, Jennifer RS Meadows, Marc P Höppner, Jacques Dainat, Henrik Lantz, Brian J Haas, Manfred G Grabherr

**Affiliations:** 1 Science for Life Laboratory, Department of Medical Biochemistry and Microbiology, Uppsala University, Uppsala, Sweden; 2 Broad Institute of MIT and Harvard, Cambridge, MA, USA

**Keywords:** Comparative genomics, Genomic coordinate translation, Genomic duplication, Cross-species gene expression analysis

## Abstract

**Background:**

Genomic duplications constitute major events in the evolution of species, allowing paralogous copies of genes to take on fine-tuned biological roles. Unambiguously identifying the orthology relationship between copies across multiple genomes can be resolved by synteny, i.e. the conserved order of genomic sequences. However, a comprehensive analysis of duplication events and their contributions to evolution would require all-to-all genome alignments, which increases at N^2^ with the number of available genomes, N.

**Results:**

Here, we introduce Kraken, software that omits the all-to-all requirement by recursively traversing a graph of pairwise alignments and dynamically re-computing orthology. Kraken scales linearly with the number of targeted genomes, N, which allows for including large numbers of genomes in analyses. We first evaluated the method on the set of 12 *Drosophila* genomes, finding that orthologous correspondence computed indirectly through a graph of multiple synteny maps comes at minimal cost in terms of sensitivity, but reduces overall computational runtime by an order of magnitude. We then used the method on three well-annotated mammalian genomes, human, mouse, and rat, and show that up to 93% of protein coding transcripts have unambiguous pairwise orthologous relationships across the genomes. On a nucleotide level, 70 to 83% of exons match exactly at both splice junctions, and up to 97% on at least one junction. We last applied Kraken to an RNA-sequencing dataset from multiple vertebrates and diverse tissues, where we confirmed that brain-specific gene family members, i.e. one-to-many or many-to-many homologs, are more highly correlated across species than single-copy (i.e. one-to-one homologous) genes. Not limited to protein coding genes, Kraken also identifies thousands of newly identified transcribed loci, likely non-coding RNAs that are consistently transcribed in human, chimpanzee and gorilla, and maintain significant correlation of expression levels across species.

**Conclusions:**

Kraken is a computational genome coordinate translator that facilitates cross-species comparisons, distinguishes orthologs from paralogs, and does not require costly all-to-all whole genome mappings. Kraken is freely available under LPGL from http://github.com/nedaz/kraken.

## Background

An organism’s genome contains a collection of genes and regulatory elements that are located in particular order and orientation. The organization and relative distance of these features from one another can direct their activity patterns. A classic example includes the Hox gene clusters [1,2], which are preserved as four to eight distinct regions among vertebrates [3]. The Hox proteins and their regulatory machinery control organism development, and while the different copies share sequence similarity with each other, it is the spatial organization that determines the timing of expression. The Hox genes have undergone several duplication and expansion events during evolutionary history, allowing for more specialized roles in fine-tuning development, facilitating increased organismal complexity [4]. More generally, gene duplications are a common mechanism for subsequent sub- and neo-functionalization, both in terms of proteins, as well as in regulation. For example, up to two thirds of vertebrate genes are members of families [5], which have been generated by local duplications, block duplications and whole genome duplications.

For a comprehensive comparative genomics study, it is thus paramount to accurately identify orthologous sequences that arose through duplication events, both prior to and after speciation. Due to complex patterns of selective pressure, resulting in conservation of sequences, loss of sequences, as well as invention of new functions, nucleotide or protein similarity is not a reliable measure to unambiguously resolve orthologs [6]. However, orthologs can be recognized through conserved synteny, i.e. the locally conserved order and orientation of features, which has been observed even in species with high turnover rates of gene duplication, expansion and loss, such as in mammals [7]. Synteny alignments are either computed relative to one central genome, as for example human, as described in the 29 mammalian genomes project [8], or via a complete set of pairwise comparisons, where the computational time for the analysis of N genomes is in the order of O(N^2^). Here, we describe a novel computational method, Kraken, which provides both the independence of a central genome, as well as eliminating the time consuming step of generating all pairwise synteny maps. For setting up a synteny framework, Kraken uses maps generated by the genome-wide synteny alignment programs and methods such as LASTZ chained alignments [9], Mummer [10], SyMAP [11], Satsuma [12], SynMap [13], or alignment graphs, for example Enredo/Pecan [14], and HAL [15]. Next, Kraken infers indirect syntenic relationships between two genomes not connected through a synteny map via indirection, i.e. the mapping of regions through a graph of synteny maps and by dynamically augmenting local alignments.

As such, Kraken constitutes a core utility aiming at resolving several bioinformatics challenges currently facing life sciences. In particular, high-throughput sequencing technologies generate millions of reads from RNA, which, in turn, predict tens or hundreds of thousands of transcribed loci, each of which can contain multiple isoforms. To assess the quality and biological relevance of each prediction, additional evidence is required. An automated utility, which compares transcriptional activity in orthologous regions across multiple species, can provide such evidence, in particular for novel loci that might have a function that is yet to be determined.

In the following sections, we describe Kraken’s implementation, and evaluation of the accuracy, sensitivity, and specificity on a set of 12 *Drosophila* genomes spanning a long range of genomic distances [16] and the well-annotated mouse, human, and rat genomes. We then apply Kraken to rapidly and automatically establish transcription orthology maps across multiple vertebrate species for known and novel loci, leveraging previously generated RNA-Sequence data [17].

## Implementation

**Figure 1:**
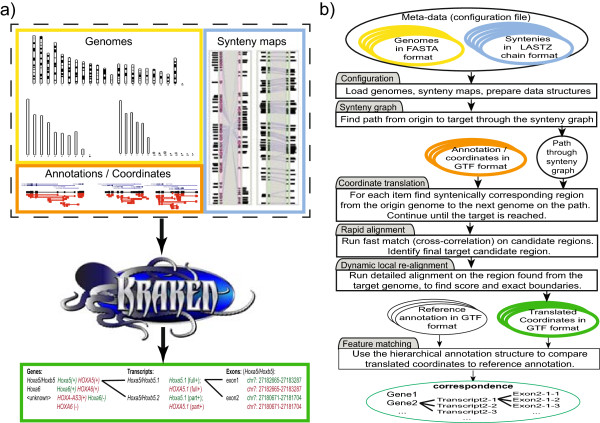
**Overview of Kraken. (a)** Showing Kraken’s input and output: genomes are supplied in FASTA format, synteny maps are in LASTZ general format, computed by any synteny based aligner such as LASTZ chained alignments [9], Satsuma [12], SyMAP [11], Enrodeo/Pecan alignment graphs [14]. Annotations are provided in Genome Transfer Format (GTF) files for one or more genomes, and can be either curated annotations, or experimentally found sequences, e.g. through Tophat/Cufflinks [18] or Trinity/PASA [19]. As output, Kraken lists the input GTF file items in output genome coordinates, as well as spatial relationships between translated features and features that are native to the genome. Features are hierarchically organized into loci, which are sets of transcripts, which are collections of exons. **(b)** Technical overview of Kraken’s workflow. Genomes and synteny maps are loaded, and a complete set of paths connecting each genome to each other is computed prior to processing. For each annotation, Kraken finds an exact or approximate orthology match in each other genome, and runs a two-step local alignment to determine the boundaries of the orthologous feature. Once completed, Kraken examines the locus-transcript-exon structure of the translated input and compares it, if available, to annotations native to the target genome.

An overview of Kraken’s workflow is shown in Figure 1a: the input is comprised of (i) genome sequences, in chromosome or scaffold coordinates; (ii) synteny maps, generated from synteny alignment programs or extracted from multiple alignment graphs; and (iii) annotations or feature coordinates, specified in Gene Transfer Format (GTF). For each genome, Kraken’s output is provided on two levels: (i) GTF files in translated coordinates, preserving all of the original information; and, if applicable, (ii) a list of spatial relationships between overlapping translated annotations and native annotations. Figure 1b shows a flow chart of how the input data is processed. After all genomes, synteny maps, and input coordinates are loaded into memory, each query coordinate is translated into the genomic coordinate system of the target genome in a multi-step process: (i) estimate candidate locations of orthologous coordinates through the synteny graph; (ii) perform a rapid alignment of the input sequence against the target region based on a cross-correlation algorithm; and (iii) compute a local sequence alignment to determine the exact target coordinates. Optionally, (iv) coordinates in target coordinates are compared against a reference GTF, accommodating for features with multi-exonic structures and multiple isoforms. In the following, we describe each step in detail.

### Configuration

Kraken reads the meta-data accompanying a dataset from a configuration file. This file lists the file locations of the genomes (FASTA format), the synteny maps (LASTZ general format and Satsuma format, Kraken provides conversion tools for other formats) and the genomes they connect and in what direction. Kraken requires the pre-computed pairwise synteny maps to provide the coordinates of orthologous regions (i.e. not the synteny alignments), in intervals specifying the corresponding: (i) chromosome or scaffold name; (ii) first nucleotide position in the interval; (iii) last nucleotide position in the interval. Maps need to be provided in one direction (genome A → genome B) only, and are duplicated and inverted in memory to supply both directions (genome B → genome A). All positions (start and stop) in the synteny maps are sorted by chromosome first and position next in target coordinates, setting up a data structure for binary search.

### Synteny graph

Kraken performs an exhaustive search through all possibilities from source to target genomes through a synteny graph built from pairwise synteny as specified in the configuration file, and selects the path with either (a) the lowest number of indirect mappings, or (b) by minimizing the accumulated genomic distances, if specified. The path is determined prior to coordinate translation and fixed from there on. More formally, the synteny graph G(V,E) is an undirected graph, where the nodes, V(G), consist of the genomes and the pairwise synteny alignments between the genomes constitute the edges of the graph, E(G). The graph edges are weighted by genomic distances if available, and otherwise they are non-weighted. For a given pair of genomes, (u, v), the shortest path is found by minimizing the edge distances on the path.

### Coordinate translation

Each interval specified in the source GTF is translated individually by looking up the lower bound of the start and higher bound of the end position. The coordinate start and stop can either directly fall into syntenic anchors, or in between anchors, in which case the candidate interval is widened to the next adjacent syntenic map entries. This process is repeated until the target genome is reached. Translated target coordinates on the same chromosome or scaffold with syntenic flanks in consistent orientation of up to 100,000 nt are passed on to the next step, otherwise, the region is split into two target intervals, one from each side (50,000 nt each) to allow for searching the boundaries of syntenic breaks.

### Rapid alignment

For computational efficiency, Kraken performs a quick search of the source sequence against the target interval by employing an approximate cross-correlation alignment, as originally implemented by Satsuma [12]. To limit the processing time and memory requirements of the underlying Fourier Transform, the target interval is broken into overlapping sequences of 2^14^ nucleotides in size, the source sequence is cross-correlated against each block, and the block with the highest absolute cross-correlation signal is computed. Based on the size of the source interval, Kraken determines a candidate region equal to that size plus flanks on each end (+/− 12 nt per default) based on the offset of the cross-correlation signal.

### Dynamic local re-alignment

Detailed alignment of the source sequence is performed using the Cola [20] implementation of a banded alignment with gap-affine penalties [21,22] against the target sub-sequence, which is defined by the offset determined by the rapid alignment. For source intervals of >100 nt, the sequence is split into two 100 nt chunks at each end covering the start and end region of the sequence, both in the source and the target genomes. A p-value threshold to accept alignments is configurable and defaults to 10^−4^. Alignments are not required to cover the entire source sequence, i.e. nucleotide mismatches at the boundaries are permissible (as forcing alignments would infer the risk of false insertions). In that case, the final translated target coordinates are estimated based on the alignment offset into the source sequence, i.e. if the alignment starts at position k in the source feature, the target start coordinate is adjusted by -k nucleotides (and vice versa for the final stop coordinate). Output is provided in GTF format, where for all the items that were successfully translated an output entry is produced containing the translated coordinates.

### Feature matching

Optionally, Kraken allows for directly comparing translated coordinates to features specified in the coordinates of the target genome, following the GTF file convention for exons, transcripts, and genes. Kraken stores GTF coordinates internally in the following data structures (classes): (i) exons, which are intervals (implemented as annotation items), consisting of a chromosome name, start and end coordinate; (ii) transcripts, which are generalizations of items and extend functionality by owning a number of exons; and (iii) loci, which also generalize items but own a set of transcripts. Kraken instantiates arrays of each type in sorted order for efficient retrieval, and stores ownership relationships between items, transcripts and loci bi-directionally to facilitate fast referencing in either direction. Thus, Kraken allows for rapidly inferring spatial relationships between the genomic features in the source and target genome, if the latter are available in GTF format, and taking into account their multi-exonic structures. Kraken classifies matches as: (i) full sense overlap, i.e. all exons of the source transcript overlap all exons of the target transcript and fall on the same strand; (ii) partial sense overlap, i.e. one or more exon overlap in sense direction; (iii) intronic (sense or antisense), i.e. the coordinates of the source transcript overlap an intron of a target locus; and (iv) antisense (full or partial), i.e. overlapping target exons in the opposite strand. The coordinates of all translated features, the relationships described above, and the overlapping target annotations are reported in human-readable outputs that are also friendly to machine parsers.

## Results and discussion

### Translating genomic intervals between 12 fruit fly species: evaluating synteny graphs

**Figure 2:**
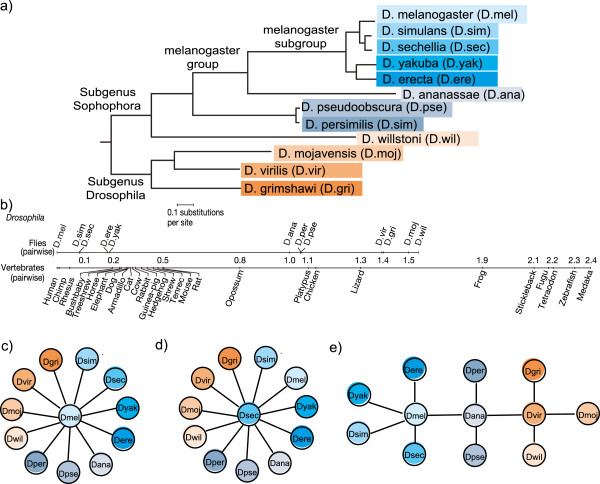
**Synteny topologies for indirect feature mappings in 12 *drosophila *species. (a)** The phylogeny of 12 *Drosophila* species is shown, as inferred by [16], and **(b)** a comparison of evolutionary distances between fruit flies and vertebrates. **(c)** Shown is a Kraken star configuration with D.melanogaster, the most complete genome, in the middle, where indirect translations are forced to map through the D.melanogaster genome. **(d)** Alternatively, a star configuration centered around D.sechellia is shown, as well as **(e)** a more complex configuration loosely modeled on species phylogeny and with three genomes serving as transition points.

To examine how translation through intermediate synteny maps along a graph impacts sensitivity, we evaluated Kraken on 264 pairwise comparisons between the genomes of 12 *Drosophila* fruit flies [16]. *Drosophila* species are a diverse group (Figure 2a) and cover small to large genomic timespans (Figure 2b), allowing for measuring accuracy as a function of genomic distance. Moreover, the genomes are of moderate size (~150 Mb), so that computing a full set of pairwise synteny maps as the baseline for comparison is computationally feasible. We first generated all pairwise genome-wide syntenic alignments using Satsuma [12]. We next selected 75,000 random genomic intervals of 200 base pairs in size for each genome, and used Kraken to translate these sequences into the coordinates of all other genomes using three different topologies: (i) a star configuration with D.melanogaster in the center (Figure 2c); (ii) a star configuration with D.sechillia in center (Figure 2d); and (iii) a clade configuration (Figure 2e) roughly following species phylogeny (Figure 2a). Table 1 summarizes the results. We first observed that for closely related species, such as D.yakuba and D.erecta, more than 80% of blocks could be successfully translated, while this fraction drops to about 12% for more distantly related species such as D.simulans and D.wilstoni. However, genomic distance does not majorly impact the fraction of successful indirect to direct translations: for example, for the closely related species pair D.erecta/D.yakuba in the melanogaster star configuration, 97.9% of directly translated blocks are matched in the indirect translation, while this fraction is only slightly lower for the more distantly related pairs D.erecta/D.mojavensis (96.9%) and D.erecta/D.willstoni (95.5%). The median fraction of identical indirect/direct translations is highest in the D.melanogaster star topology (Figure 2c), followed by the D.sechellia star (Figure 2d), and clade (Figure 2d) topologies. While overall, using the high-quality genome of D.melanogaster as the center yields the best results with respect to the topologies evaluated here, individual statistics suggest that a more complex topology allowing for alternative paths to traverse the synteny graph yields higher sensitivity (Table 1).

**Table 1:** **Comparison of direct and indirect pairwise coordinate translations for the 12-*Drosophila *** **dataset**

**Source**	**Target**	**Total blocks**	**Direct mapped**	**% of Tot mapped**	**Melanogaster Star Configuration**		**Sechellia Star Configuration**		**Clade center configuration**	
					**Mapped**	**Ident. (%)**	**Mapped**	**Ident. (%)**	**Mapped**	**Ident. (%)**
D.ana	D.ere	52808	23003	43%	22923	99.0	22744	98.3	22923	99.0
	D.gri	53157	7889	16%	8498	96.3	7775	89.9	8083	93.5
	D.moj	53000	8316	16%	8362	95.9	7648	88.1	8122	93.2
	D.sim	53012	21720	41%	21975	98.7	21766	98.2	21975	98.7
	D.wil	53435	8011	16%	8048	92.8	8056	93.1	7424	86.2
	D.yak	53375	23168	43%	23065	98.3	22898	98.7	23065	98.3
D.ere	D.gri	61131	9121	16%	9536	96.9	8743	90.6	8607	87.5
	D.moj	60856	9341	15%	9238	96.9	8395	87.3	8373	85.9
	D.per	60645	16423	27%	16288	98.0	16296	97.0	15898	95.3
	D.pse	61162	17539	28%	17459	98.6	17337	97.2	17115	96.0
	D.sim	61335	46231	76%	46011	98.4	45936	98.3	46011	98.4
	D.vir	60926	9928	16%	9713	95.0	9816	95.6	9324	90.1
	D.wil	60704	8628	14%	8528	95.5	8535	95.4	7420	80.5
	D.yak	61272	51039	83%	50318	97.9	50244	97.8	50318	97.9
D.gri	D.moj	61933	22084	35%	20200	90.8	18586	83.4	22109	98.9
	D.per	62029	8393	14%	8843	94.7	8068	88.6	8160	88.1
	D.pse	61852	9328	15%	9325	94.0	8476	86.5	8675	87.6
	D.sim	61844	8038	13%	8455	97.7	7624	89.8	7701	88.8
	D.wil	62007	7638	13%	7380	88.1	6860	83.4	7586	93.4
	D.yak	61729	8760	14%	8935	97.5	8112	89.7	8180	88.7
D.moj	D.per	67626	8068	13%	8403	94.4	7752	88.1	7844	88.8
	D.pse	67781	9001	13%	8901	94.7	8141	87.3	8251	88.2
	D.sim	67662	7831	12%	8153	96.5	7430	89.7	7528	89.6
	D.wil	66834	7462	12%	7314	87.7	6870	84.4	7456	93.0
	D.yak	67142	8580	13%	8721	95.8	7887	88.9	8068	88.7
D.per	D.sim	29281	8133	28%	8181	97.7	8093	97.1	8140	97.1
	D.vir	29314	5133	18%	4897	91.9	4935	92.1	4871	91.3
	D.wil	29300	4635	16%	4420	89.0	4382	88.8	4079	81.8
	D.yak	29451	8534	29%	8478	97.6	8439	97.4	8403	96.6
D.pse	D.sim	59107	15465	27%	15776	97.7	15596	97.0	15592	96.6
	D.vir	59041	9857	17%	9346	91.8	9393	92.1	9249	90.6
	D.wil	59337	8938	15%	8582	90.2	8546	90.2	7757	81.8
	D.yak	59277	16671	28%	16545	97.6	16474	97.3	16387	96.6
D.sim	D.vir	65022	8855	14%	8829	95.9	8867	93.6	8437	90.5
	D.wil	64973	7943	12%	7921	95.3	7862	92.5	6864	80.1
	D.yak	64934	46720	72%	45819	97.2	46188	96.5	45819	97.2
D.vir	D.yak	63073	8988	14%	8781	93.5	8851	93.6	8649	91.4
D.wil	D.yak	48969	6204	13%	5684	87.5	5748	87.8	5061	76.8
Median Mapping Ratio (%)						97.4		93.6		91.7

Each row shows results for blocks of 200 nucleotides randomly chosen from the source genome. Results are shown for three different configurations for indirect translation that are depicted in Figure 2, namely, D.melanogaster-star, D.sechellia-star and the clade-center configuration, where in each case we report two items: (i) total number of blocks translated through the specified configuration (indirect) and (ii) the fraction of directly translated blocks that are matched by identical indirect translations. To compact the results, pairwise comparisons where at least one configuration would result in a direct comparison have not been included here. Also, the species are shown in abbreviated format; see Figure 2 for equivalent complete species name.

### Matching genes between human, rat, and mouse: a quantitative assessment

**Figure 3:**
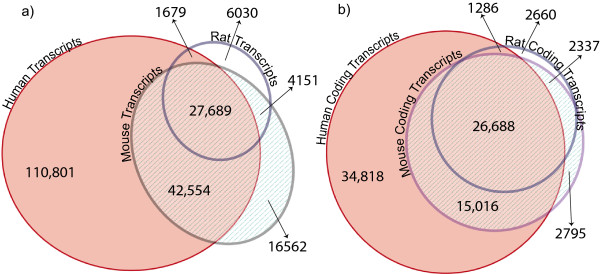
**Venn diagrams of transcript overlaps between human, mouse, and rat. (a)** All annotated transcripts, which include all isoforms of genes, are shown for each species in circles. Two-way and three-way spatial matches between transcripts in different species are shown as overlaps in the middle, counting only maximal matches (isoforms for which the largest number of exons overlap across genomes). In human, the number of annotated isoforms is about two-fold higher than in mouse and four-fold higher than in rat. **(b)** Counts and cross-species overlaps of transcripts from only protein coding loci are shown.

We evaluated Kraken’s quantitative performance with regards to known genomic features by matching translated gene structures across three well-annotated high-quality mammalian genomes: human (hg19), mouse (mm10), and rat (rn5). Using pairwise synteny maps between the genomes generated by LASTZ [9], we translated all annotated genes (Ensembl 68) in six pairwise comparisons across genomic coordinates. Table 2 summarizes the results: overall, between 77% and 95% of transcripts could be unambiguously translated, with between 90% and 96% of these overlapping with annotated loci in the target genome. This fraction is higher for the protein-coding set, ranging from 93% to 98%, likely due to higher sequence conservation and sequence similarity. Kraken matched more annotated transcripts between human and mouse than human and rat, possibly because of differences in the quality of the genome builds and/or annotation. Among the 1,760 protein coding gene loci that failed to be translated from human to mouse, more than half (926) can be divided into the following five categories: (i) 483 uncharacterized and hypothetical proteins; (ii) 188 loci with predicted open reading frames but without functional prediction; (iii) 113 pseudogenes from ribosomal proteins; (iv) 56 zinc finger proteins; (v) 51 olfactory receptors; and (vi) 35 keratin associated proteins. While the first two categories represent genes of uncertain annotation status, the latter are comprised of members of gene families that are highly variable in copy number, or genes that have been known to undergo lineage-specific expansions. Figure 3 shows three-way Venn diagrams based on maximal overlaps (i.e. one-to-one relationships between transcripts in different genomes) for all transcripts (Figure 3a), and for the protein-coding subsets (Figure 3b). As in the pairwise comparisons, the fraction of overlaps is higher for protein coding genes. Notably, the rat annotates the lowest number of isoforms per protein coding gene, however, more than 90% of those overlap isoforms in both human and mouse, and almost all overlap mouse transcripts, suggesting that the rat annotation mostly contains dominant isoforms. We attribute the large fraction of ‘human-only’ transcripts to the more than three-fold number of annotated alternative isoforms per human gene locus.

**Table 2:** Results of translating transcripts between the human, mouse, and rat genomes shown for all transcripts and separately for coding transcripts only

**Target source**		**Human**	**Mouse**	**Rat**
Human	All Transcripts Mapped		152237 (83.3%)	148655 (81.4%)
	All Transcripts With Overlap		141702 (93.1%)	135324 (91.0%)
	Coding Transcripts Mapped		75531 (97.1%)	74238 (95.4%)
	Coding Transcripts With Overlap		74356 (98.4%)	72069 (97.1%)
Mouse	All Transcripts Mapped	73448 (80.7%)		86420 (95.0%)
	All Transcripts With Overlap	70243 (95.6%)		75606 (89.4%)
	Coding Transcripts Mapped	42506 (90.7%)		45802 (97.8%)
	Coding Transcripts With Overlap	41704 (98.1%)		43483 (94.9%)
Rat	All Transcripts Mapped	30396 (76.8%)	35361 (89.4%)	
	All Transcripts With Overlap	29368 (96.6%)	31840 (90.0%)	
	Coding Transcripts Mapped	28754 (87.2%)	31313 (94.9%)	
	Coding Transcripts With Overlap	27974 (97.3%)	29025 (92.7%)	
	All Transcripts Total	182723	90956	39549
	Coding Transcripts Total	77808	46836	32971

For each comparison instance we show two items: (i) the total number of transcripts that Kraken successfully translates and (ii) the number of successfully translated transcripts for which we find an exon overlap based on comparison with a reference annotation. We also show in brackets: (i) the percentage of translated transcripts to the total number of transcripts and (ii) the ratio of overlapping transcripts to the total number translated.

We next compared the results to the methods HalLiftover [15] and RATT [23], using the Ensembl Compara database [24], a manually curated set of orthologous relationships between protein-coding transcripts across species for human, as independent metrics (Table 3). All methods largely agree in their predictions, with Kraken consistently and on all data sets exhibiting the highest overlap with ComparaDB, as well as the lowest number of transcripts predicted by ComparaDB only (Table 3). We note that RATT, which we ran in ‘species mode’ [23], yielded similar results to HalLiftover and Kraken on the mouse/rat comparison, but considerably fewer predictions for the more distantly related human/mouse and human/rat data sets.

**Table 3:** Results of comparing the Human-Mouse-Rat transcript overlaps with Ensembl ComparaDB orthologs for Kraken, HalLiftover, and RATT

		**Common in both**	**Unique to ComparaDB**	**Not in ComparaDB**
Human to mouse	Kraken	124733	5683	19378
	HalLiftover	122754	7662	26009
	RATT	15665	114751	2337
Human to rat	Kraken	118419	6869	19233
	HalLiftover	117869	7419	28859
	RATT	14494	110794	2114
Mouse to rat	Kraken	66667	2172	10624
	HalLiftover	61337	7502	9033
	RATT	61127	9394	7712

The numbers in each row left to right show (i) the overlap, i.e. transcripts predicted by both ComparaDB and the methods Kraken, HalLiftover, and RATT respectively; (ii) transcripts found only in ComparaDB; and (iii) transcripts found by Kraken, HalLiftover, or RATT, but not ComparaDB.

### Accuracy on nucleotide level: a qualitative assessment

**Figure 4:**
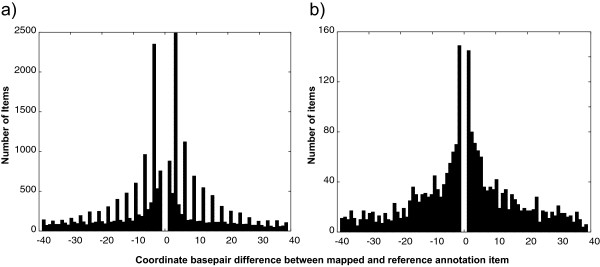
**Histogram of nucleotide differences between native mouse annotations and predictions from human. (a)** After excluding exact matches between the native mouse annotations and exon coordinates predicted from translated human annotations, the differences for coding genes show a pronounced periodicity of multiples of three nucleotides, consistent with differences in amino acid counts between species, rather than mapping or alignment errors. **(b)** By contrast, non-coding RNAs do not show any periodicity, consistent with these sequences not being subject to translation and thus free of constraint to preserve multiplicity.

We next examined the accuracy of Kraken, comparing the translated boundaries of exons to gene models native to the target genome for the human, mouse, and rat dataset (Table 4). For protein coding exons, Kraken matches 70 to 83% of complete exons between genomes with exact agreement at both splice junctions, and up to 97% of exon with exact matches in at least one splice junction. In examining cases in which the predictions did not exactly match the annotations, we found that the majority of differences differ by multiples of three nucleotides (Figure 4a), which is consistent with varying numbers of amino acids across species, indicating that the predicted coordinates could reflect actual biological differences in transcription between species. By contrast, we did not observe this pattern for exons of long intergenic non-coding genes (Figure 4b), which further supports that the periodicity observed for protein coding genes is biologically valid, rather than stemming from algorithmic artifacts, such as systematic alignment biases.

**Table 4:** Data demonstrating precision of translation at nucleotide level, shown for coding sequence, between human, mouse, and rat

**Target source**		**Human**	**Mouse**	**Rat**
Human	Total Items Mapped		241261	225012
	Exactly Matched Items		174432 (72.3%)	156654 (69.6%)
	Exactly Matched at least One Side		231333 (95.9%)	214390 (95.3%)
Mouse	Total Items Mapped	201649		200606
	Exactly Matched Items	157304 (78.0%)		166079 (82.8%)
	Exactly Matched at least One Side	188982 (93.7%)		195357 (97.4%)
Rat	Total Items Mapped	174516	180701	
	Exactly Matched Items	132835 (76.1%)	148839 (82.4%)	
	Exactly Matched at least One Side	160294 (91.9%)	171099 (94.7%)	

For each comparison three items are shown: (i) total number of coding sequences translated from source to target species, (ii) number of items translated that also find an exact match in the reference target annotation (iii) similar to ii but also including those items that exactly match either on their start or stop coordinate. For (ii) and (iii), the percentage of category items matched to the total number mapped is given in brackets.

### Resolving transcribed single-copy genes and gene family orthologs in vertebrates

**Figure 5:**
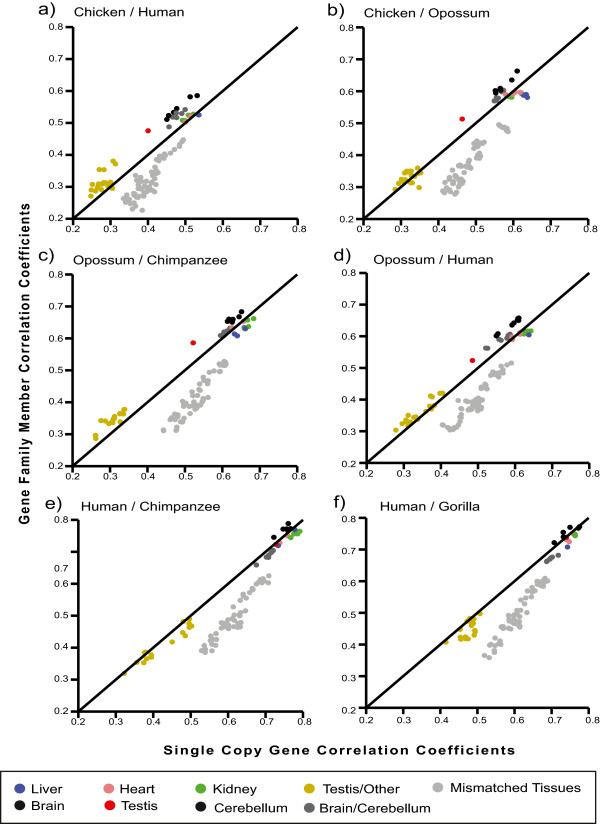
**Correlations of single-copy genes and gene family members.** We show scatter plots comparing tissue-specific correlations of single-copy genes and gene family members on the species pairs: **(a)** chicken versus human, **(b)** chicken versus opossum, **(c)** opossum versus chimpanzee, **(d)** opossum versus human, **(e)** human versus chimpanzee, and **(f)** human versus gorilla. In all panels, Spearman’s correlation is shown on the x-axis for single-copy genes, and on the y-axis for gene families. Tissue comparisons are coded by color (see legend), in all cases, brain tissues (labeled as ‘brain’ and ‘cerebellum’ are more correlated for gene families (black dots), while mismatched-tissue comparisons, with the exception of the brain and cerebellum tissues, are more correlated for single copy genes (light grey dots).

In order to illustrate Kraken’s power as a practical tool for processing large and complex datasets, we re-analyzed publicly available RNA-Sequence data obtained from different vertebrates and multiple tissues [17]. We first mapped the RNA-Sequence reads onto the respective genomes of human, chimp, gorilla, opossum, and chicken using Tophat [18], without reference annotation guidance, and allowing for the detection of un-annotated transcripts and isoforms. Next, we estimated RPKM expression values from the unpaired reads using Cufflinks/Cuffdiff [18] and merged experimentally found transcripts with the reference annotations (Ensembl 64) using Cuffmerge [18]. We then used Kraken to translate the coordinates of transcribed features through a minimal set of LASTZ-chained alignments. A selection of pairwise interspecies transcript relationships are summarized in Table 5: overall, the number of transcripts that can be translated decreases with genomic distance from 250,000 for human-chimp to 100,000 for chicken-human. Likewise, the fraction of protein-coding genes, which are among the most highly conserved genomic features, is higher for more distantly related organisms. We next investigated whether protein-coding single copy genes and gene family members, defined by Ensembl [5] based on clustering by protein similarity, consistently show distinct expression patterns across tissues and species (we excluded genes with RPKM values < 1). We computed Spearman’s correlation coefficients between the tissues of all species-pairs separately for the two gene sets, and compared them to each other, highlighting the differences (Figure 5): in all comparisons of mismatched tissues (e.g. brain versus liver), single copy genes are more correlated than orthologous gene family members (light grey dots, Figure 5). Moreover, gene family paralogs are more highly correlated in matched brain and cerebellum tissues compared to single copy genes (black dots, Figure 5), while liver and kidney show the opposite trend (green and blue dots, Figure 5), with heart showing patterns in between (pink dots, Figure 5). In all cases, testis is least correlated compared with other tissues (yellow dots, Figure 5) both in single copy genes and gene families, and for large genomic distances (chicken-human, chicken-opossum, opossum-human). Testis genes are also less correlated in cross-species comparisons, while correlation is comparable to other tissues in primates (red dots, Figure 5). In terms of absolute values for correlations of both single copy and multiple copy genes, species are grouped according to phylogeny, including the primate clade, albeit statistically weakly (p < 0.086), with 10 out of 15 matched tissue comparisons (including male/female pairs) placing human closer to chimp than gorilla. Notably, comparisons involving kidney indicate higher correlations for human-gorilla than human-chimp.

**Table 5:** Pairwise interspecies transcript relationships based on analyzing RNA-Sequence data generated and published by Brawand et al. 2011

**Species in comparison**		**Total Source transcripts**	**Mapped transcripts w/hit**	**Ensembl transcripts**	**Protein coding transcripts**	**Singleton transcripts**	**Singleton loci**	**Families transcripts**	**Families loci**
**Source**	**Target**								
Chicken	Human	100823	51492	50278	49944	13149	3968	36795	8857
	Opossum		49500	48535	48271	12452	3702	35819	8497
	Chimp		47357	46466	46212	11786	3514	34426	8233
	Gorilla		48585	47604	47326	12146	3632	35180	8420
Opossum	Human	162989	80994	78492	77581	16936	4246	60645	12611
	Chimp		75338	73622	72979	15960	3979	57019	11581
	Gorilla		76486	74620	73935	16227	4049	57708	11837
Human	Chimp	253887	211785	205096	180964	41267	5486	139697	16234
	Gorilla		216536	210180	183659	41743	5588	141916	16498

For each source-target genome pair, we list the number of transcripts mapped from the source that overlap with annotations in the target, indicating the number of annotated transcripts, and the number annotated as protein coding genes in the target genome. We further list overlaps of single-copy genes and gene family members, determined by the genome of the source, with annotated protein coding genes in the target both at the transcript and locus level.

### Expression of novel transcripts in three primates

**Figure 6:**
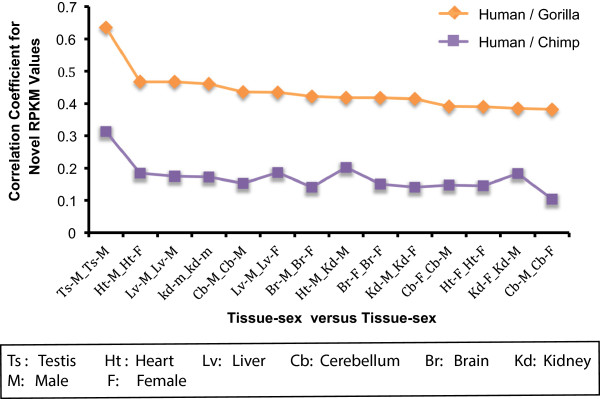
**Correlation of un-annotated transcribed features in human, gorilla, and chimpanzee.** We show the Spearman’s correlation between RPKM expression values across tissues of novel transcribed features between human versus chimp (purple) and human versus gorilla (orange), ranked by correlation in the latter set. Overall, novel transcripts are more highly correlated between human and gorilla, with, in both cases, testis being the most highly correlated tissue, followed by heart, liver, and kidney. The sex of the tissue donors was not found to be statistically significant.

Kraken’s independence of known annotations or protein coding genes allows for the analysis of transcribed loci that have not previously been characterized. For example, thousands of long intergenic non-coding RNAs have recently been found in human [25], mouse [26], zebrafish [27], and dog [28]. In the human genome, we found a total of 67,580 actively transcribed loci based on RNA-Seq data [17], 17,421 of which fell outside of Ensembl annotations. Of these, 6,281 and 5,968 were also actively transcribed in chimpanzee and gorilla respectively, with 3,503 transcribed in all three genomes. While the expression levels are correlated across species at much lower levels than those of known protein-coding genes, matched tissue comparisons still exhibit statistically significant correlations (Figure 6), with testis being the most correlated tissue in both human-chimp and human-gorilla. The reported correlation measures have high statistical significance with a maximum p-value of 10^−8^.

## Conclusions

The analytical power of comparing features across multiple genomes has been demonstrated in the past and dates back to the early days of modern genomics. More recent examples, in which the order and orientation of genomic landmarks played a critical role, include describing a whole genome duplication event in the baker’s yeast *Saccharomyces cerevisiae*[29], and several analyses of evolution in the chordate and vertebrate lineages (e.g. [30-33]). Recent advances in DNA sequencing and assembly, coupled with the generation and processing of functional sequence datasets, notably RNA-Sequencing, which often result in hundreds of thousands of transcripts, highlight the need for a new generation of high-throughput computational analysis tools. Such tools are required to process: (i) large genomes; (ii) large numbers of genomes; (iii) large sets of genomic features, including large numbers of paralogous loci; but: (iv) without large computational efforts. Here we described Kraken, a novel computational method and software that fills this niche. Using the genomes of 12 fruit flies, we showed that the ability to translate indirectly, i.e. use an intermediate genome as a guide and then re-computing local alignments, comes at marginal cost in sensitivity, but at a substantial gain in computational efficiency: by removing the need to statically compute all-to-all synteny maps, Kraken scales linearly with the number of genomes and allows for the simultaneous analysis of dozens or even hundreds of genomes. We next evaluated Kraken on the well established and highly scrutinized genomes of human, mouse, rat, and showed that mapping orthologous sequences is highly accurate in predicting the precise boundaries of genomic features. This is particularly relevant for comparing protein-coding genes, since errors of even a single nucleotide can cause erroneous frame shifts and stop codons in open reading frames. Thus, Kraken can be used with ease to either create or improve comprehensive annotations through orthology for genomes that have little or no validated evidence within a few CPU hours, a functionality that is also provided by other, albeit more specialized software programs, such as RATT [23], GeneMapper [34], and HalLiftover [15].

To showcase Kraken’s utility for large-scale research projects aimed at discovering hitherto unexplored functional connections between orthologous members of gene families residing in different genomes, we re-analyzed a previously published large and comprehensive dataset consisting of RNA-Seq data from different tissues and multiple vertebrates [17]. We emphasize that Kraken completed the analysis presented here within only a few hours of wall clock time and with minimal human involvement, yet yielding a rich set of results that are concordant with biological expectations and previous reports. In summary, we found that single-copy genes are more highly correlated across tissues and species than gene family members, which would indicate that single copy genes are enriched for fundamental cellular function essential to cells of different tissue types. By contrast, paralogs of gene families could have taken on more specialized roles, consistent with the concept of duplication and subsequent neo- and sub-functionalization [35]. Moreover, orthologous gene family members are more highly correlated in brain tissues across species than their single-copy gene counterparts. This suggests that a number of genes, which were duplicated early in the vertebrate lineage, took on specialized functions in the brain, and were subsequently fixed in expression patterns, whereas we did not observe this consistently in the other tissues for which sequence was available. The pattern we see would fit with the theory that the complexity of the vertebrate brain and nervous system can in part be contributed to the redundancy of genes following the two whole genome duplications early in vertebrate evolution [36,37]. Moreover, in three primates we found overlap of un-annotated transcribed regions, likely non-coding RNAs, with transcription levels most highly correlated across species in testis. Testis has been known to be the most actively transcribed tissue [38], and our results suggest that transcription does not occur in a random fashion. Long non-coding RNAs have recently been shown to take part in the circuitry controlling stem cell pluripotency and cell differentiation [26], consistent with expression in reproductive tissues, and indicating that the set of known RNAs is still an incomplete subset of the full inventory of all such transcribed loci.

In conclusion, we expect Kraken to dramatically reduce computational analysis time when deployed in future large-scale comparative studies. For a newly sequenced mammalian genome, for example, generating synteny maps to a single other mammal is sufficient for comparing it to dozens of others, at little extra computational cost. Moreover, the availability of second- and third-generation DNA and RNA sequencing technologies have made it possible to expand the set of reference genomes and expressed sequences into different branches of life. Successful and rapid analyses will thus require a computational framework that is easy to use and easy to set up.

## Availability and requirements

**Project Name:** Kraken - A Universal Genomic Coordinate Translator for Comparative Genomics

**Project home page:**http://github.com/nedaz/kraken

**Operating system(s):** Linux

**Programming Language:** C++

**License:** Source code freely available under LPGL

## Competing interests

The authors declared that they have no competing interests.

## Authors’ contribution

NZ and MGG designed and developed the software; NZ, GS, MPH, JRSM, JD, HL, BJH, and MGG designed and performed the analyses. MPH, GS, JRSM, HL, and MGG provided the biological interpretation of the results. All authors wrote the manuscript.
